# A new species of *Glyptapanteles* Ashmead (Hymenoptera, Braconidae, Microgastrinae) within *Macrobrochis gigas* (Lepidoptera, Arctiidae, Lithosiidae) in Fujian, China

**DOI:** 10.3897/zookeys.913.46646

**Published:** 2020-02-19

**Authors:** Ciding Lu, Jinhan Tang, Wanying Dong, Youjun Zhou, Xinmin Gai, Haoyu Lin, Dongbao Song, Guanghong Liang

**Affiliations:** 1 Forestry College, Fujian Agriculture and Forestry University, Fuzhou 350002, China Fujian Agriculture and Forestry University Fuzhou China; 2 Forestry Bureau of Ningde City, Ningde, Fujian, 352100, China Forestry Bureau of Ningde City Fujian China; 3 College of Forestry and Landscape Architecture, South China Agricultural University, Guangzhou, Guangdong, 510642, China South China Agricultural University Guangzhou China; 4 Baimi Biological Industry Co. Ltd., Xianning, Hubei, 440002, China Baimi Biological Industry Co. Ltd Hubei China

**Keywords:** *
Glyptapanteles
*, *Macrobrochis gigas*, new species, Oriental Realm, parasitoid

## Abstract

The south-east coastal area of Fujian, China, belongs to the Oriental Realm, and is characterized by a high insect species richness. In this work, a new species of Hymenopteran parasitoid, *Glyptapanteles gigas* Liang & Song, **sp. nov.** found in Jinjiang within hosts of caterpillars *Macrobrochis gigas* (Lepidoptera: Arctiidae), is described and illustrated, with differences from similar species. Additionally, we presumed that both parasitoid and host species play very important role in the coevolution and tritrophic interaction between plants, phytophagous insects, and their parasitoids, because these insects probably broke the sporangia and made contributions to their colonization, or some spores were spread for long distances by adult moths after their emergence, or some parasitoids were attracted by the eggs and larvae of these caterpillars, which was also thought to be helpful to spread of spores.

## Introduction

In an agricultural ecosystem, insect parasitoids play very important roles in the trophic networks and attract much research for their potential in biological control (Godfray 1994), especially as some species have largely been reared artificially and then released in the fields to suppress the pest populations (Liang et al. 2015). Each species kills and lives at the expense of host as a result of its development after ovipositing its egg within or on the surface of the host (Eggleton and Gaston 1990; Rachel et al. 2016). There are an estimated 60000 species of insect parasitoids belonging to the Hymenoptera (Lasalle and Gauld 1993), encompassing thousands of species specifically or generally attacking host eggs, larvae, or pupae of Lepidoptera (Shenefelt 1973; Clausen 1978; Shaw 1983; Chen and Wu 1994; Chen and Ji 2003; Traugott et al. 2006; Daane et al. 2013; Daciana et al. 2016), which would be alternative biological control agents to suppress populations of caterpillars. Previous taxonomic works have provided species identification and great contributions to potential agents of biological control. In this work, we described a new species of the genus *Glyptapanteles* Ashmead from the south and east coastal line (24°30'-24°54' North) in Fujian, China, emerged from its host caterpillar species *Macrobrochis gigas* (Walker, 1856) (Lepidoptera: Arctiidae) (Walker 1965). This caterpillar feeds on mosses growing on the trunks of trees.

## Materials and methods

### Field sampling

During field surveys for wasps parasitizing *M. gigas*, we discovered a new species of *Glyptapanteles* Ashmead, 1904 (Braconidae, Microgastrinae) associated with the caterpillars feeding specifically on mosses. This new species was found in the summers of 2015-2016 from Linyuan town (N24°44'40.42", E118°30'55.65") and Yonghe town (N24°42'57.34", E118°35'10.42") in Jinjiang, Fujian, south and east coastal areas of China. Braconidae parasitoids of *M. gigas* were sampled using two methods: (1) ectoparasitic cocoons of parasitoids coupled with mummies of host larvae were collected from the trunks of trees; (2) and some living larvae at different instar stages were collected every ten days and reared with mosses indoor to obtain the specimens of parasitoid species. Hymenopteran parasitoids were then sorted and identified.

Morphological diagnosis

Parasitoid adult specimens were cleared up and mounted in Canada Balsam or in Euparal® for measurement of appendages under a stereomicroscope (LEICA 205C, Germany). All figures were made using Leica Application Suite (LAS V 4.0) software. One female paratype specimen was sputter gold-coated and examined using a Jeol JSM – 6380 LV Scanning Electron Microscope. For identification of the subfamily Microgastrinae, see van Achterberg (1990, 1993); for references to the genus and other genera mentioned in this paper, see Yu et al. (2016). Morphological terminology follows van Achterberg (1988, 1993) and Eady (1974), including the abbreviations for the wing venation. TI stands for first metasomal tergite, TII for the second tergite, etc.

The types of the newly described species are deposited in the collection of the Parasitoid Wasp Museum of the Institute of Beneficial Insects, College of Plant Protection, Fujian Agriculture and Forestry University (**FAFU**), Fuzhou, China.

Abbreviations used in this paper are as follows:

**POL** Postocellar line (minimum distance between posterior ocelli)

**OD** Posterior ocellus maximum diameter

**OOL** Ocular-ocellar distance (minimum distance between posterior ocellus and eye).

## Systematics

*Glyptapanteles* was originally described in 1904 (Ashmead 1904), then segregated from *Apanteles* genus by Ashmead, but Muesebeck (1920, 1922) subsumed *Glyptapanteles* again into *Apanteles* (Whitfield et al. 2002b). Later, Nixon (1965, 1973) did not recognize *Glyptapanteles* as a valid genus in his reclassification of Microgastrinae, but finally, it was accepted as a distinct genus in 1981 (Arias-Penna et al. 2019).

*Glyptapanteles* is a cosmopolitan group of hyper-diverse parasitoid species, which occur in all faunal regions (Whitfield et al. 2018) worldwide including Australia (Austin and Dangerfield 1992), Ecuador (Whitfield et al. 2002), China (Chen and Song 2004; Zeng et al. 2007), Greece (Papp 2007), Croatia, Bosnia and Macedonia (Papp 2009), India (Sathe and Dawale 1999; Gupta and Pereira 2012; Gupta and Fernández-Triana 2014; Gupta et al. 2011, 2016), and remains taxonomically challenging worldwide due to its highly specious nature, morphological similarity amongst species and negligible host records. Recent specimens from Neotropical regions indicates this genus has the most diverse species within Microgastrinae containing *Glyptapanteles*, *Apanteles* Förster, and *Diolcogaster* Ashmead (Whitfield et al. 2018). *Glyptapanteles* has a broad habitat of ecological distribution range from 90 m to 2,800 m elevation, attacking 35 host species.

Until 2018, more than 122 species were described worldwide (Yu et al. 2016, Gupta et al. 2016), then 136 new species of *Glyptapanteles* from Costa Rica and Ecuador were described (Arias-Penna et al. 2019). Unfortunately, hundreds of species of *Glyptapanteles* remain undescribed.

The female hypopygium is evenly sclerotized from side to side, never with a series of parallel longitudinal creases. Ovipositor sheath short and mostly concealed by hypopygium, its length not more than half of the hind tibia (rarely longer, but if so, hypopygium is large and acutely pointed, concealing most of the ovipositor), sheaths dagger-shaped with only a few setae concentrated near the apex. Petiole on T1 never wider at the apex, the sides either gradually converging distally or parallel and strongly rounded to the apex. The median area on T2 broadening distally and often subtrapezoidal or truncate-trapezoidal, sometimes lateral grooves delimiting the median area are lost among many diverging aciculations and sometimes do not reach the proximal edge of T3; T3 always smooth. Propodeum usually completely or mostly smooth, but often with coarsely sculpture covering all or part of the surface; rarely with a median longitudinal carina, but never with even a trace of the areola. Fore wing with r-m vein absent, so that the small areolet is open distally. Distal half of margin of vannal lobe of hind wing convex or flattened, with or without a fringe of setae. The anterior furrow of metanotum flattened (without sublateral setiferous projections) and glabrous; scutellar phragma exposed or concealed (Mason 1981).

### Glyptapanteles gigas

Taxon classificationAnimaliaHymenopteraBraconidae

Liang & Song sp. nov.

CB56F772-92CD-572D-A80A-5FCC56F36C07

http://zoobank.org/9A8DD22D-499D-4BA2-B0B0-3504E9C12774

#### Distribution.

Linyuan town (N24°44'40.42", E118°30'55.65") and Yonghe town (N24°42'57.34", E118°35'10.42") in Jinjiang, Fujian, south and east coastal areas of China.

**Etymology.** The specific epithet is derived from the scientific name of its host *M. gigas*. Gender is masculine.

**Description.** Female (holotype). Body length 2.1 mm, fore wing length 2.3 mm.

**Figures 1–6.**
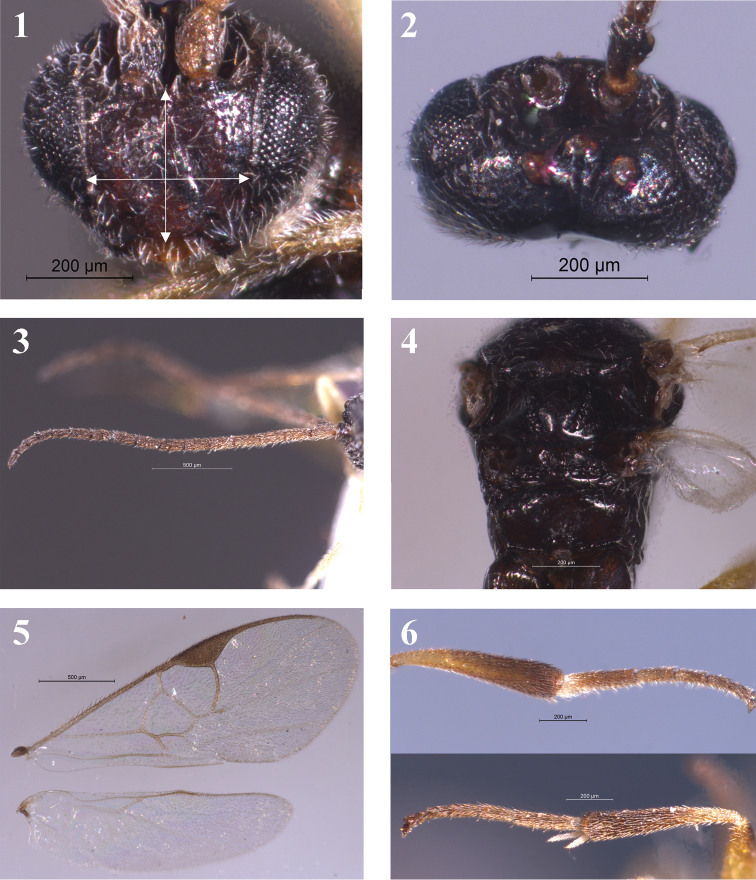
*Glyptapanteles gigas* Liang & Song, sp. nov. **1** Head approximately orbicular-ovate; face slightly convex with long hairs, FH: FW = 2.0: 2.6 **2** Head. 2.3 times as wide as long. Ocelli large, arranged in a low triangle. POL: OD: OOL= 0.7: 0.3: 0.8 **3** Antenna longer than body (10.0: 7.9); Flagellomere proportions: 2 L/W = 2.8, 8 L/W = 2.7, 14 L/W = 1.7, L 2/14 = 1.9, W 2/14 = 1.2. F12-15 tightly connected **4** Mesosoma. Note that notauli hardly exist. Propodeum relatively flat and smooth, horizontally rectangle, no median longitudinal carina **5** Wings. Forewing with areolet open; r and 2-SR meeting at a circular arc; 2-SR: r: width of pterostigma = 0.6: 0.8: 0.9. 1-CU1:2-CU1:m-cu = 0.6: 1.0: 0.7. **6** Legs. Hind coxa large, near to T3. Hind tibia approximately 0.95 times as long as hind tarsa; inner hind tibial spurs longer than outer one and about half of hind basitarsus.

Head. In anterior view, head approximately orbicular-ovate with antennal sockets slightly above middle level of eyes; face slightly convex, finely punctate associated with long hairs, ratio of FH:FW being 2.0: 2.6 (Fig. 1); eyes covered setae; inner margin of eyes slightly constricted towards clypeus. Transverse in dorsal view, 2.3 times as wide as long, 0.9 times as wide as width of mesoscutum. Ocelli large, arranged in a low triangle, posterior tangent of anterior ocellus approaching posterior ocelli. POL: OD: OOL= 0.7: 0.3: 0.8 (Fig. 2). Vertex almost smooth, with fine sparse setae; temple feebly punctate, with dense long setae; occiput smooth, slightly concave. Antenna longer than body in ratio of 10.0:7.9 (Fig. 3); flagellomeres thin, with short bristles, most flagellomeres with placodes arranged regularly in two ranks excerpt for last four or five flagellomeres. Flagellomere ratios: 2 L/W = 2.8, 8 L/W = 2.7, 14 L/W = 1.7, L 2/14 = 1.9, W 2/14 = 1.2. F12-15 tightly connected.

Mesosoma (Fig. 4). Side of pronotum with both a dorsal and a ventral carinate groove. Mesoscutum relatively flat, sparsely punctate with thin setose, relatively smooth near scutellar sulcus; notauli hardly exist. Scutellar sulcus relatively wide and deep, slightly curved; disc of scutellum smooth all over, approximately a low triangle in its shape. Propodeum relatively flat and smooth, horizontally rectangle, not inclined rear surface and no median longitudinal carina.

Wings (Fig. 5). Forewing with areolet open, vein r slightly inner oblique emitted from middle of pterostigma; r and 2-SR meeting at a circular arc and hardly distinguish from each other; 2-SR: r: width of pterostigma = 0.6: 0.8: 0.9; vein 1-R1 1.8 times as long as pterostigma, pterostigma 2.0 times as long as wide. 1-CU1:2-CU1:m-cu= 0.6: 1.0: 0.7. Hind wing narrow, vein cu-a slightly incurved, vannal lobe slightly convex with a few hairs.

Legs (Fig. 6). Slender. Hind coxa large, near to T3, compressed, almost smooth and shiny, scattered with weak granular on upper surface. Hind tibia approximately 0.95 times as long as hind tarsi; inner hind tibial spurs longer than outer one and about half of hind basitarsus. Fore distitarsus with a feeble spine.

**Figures 7–12.**
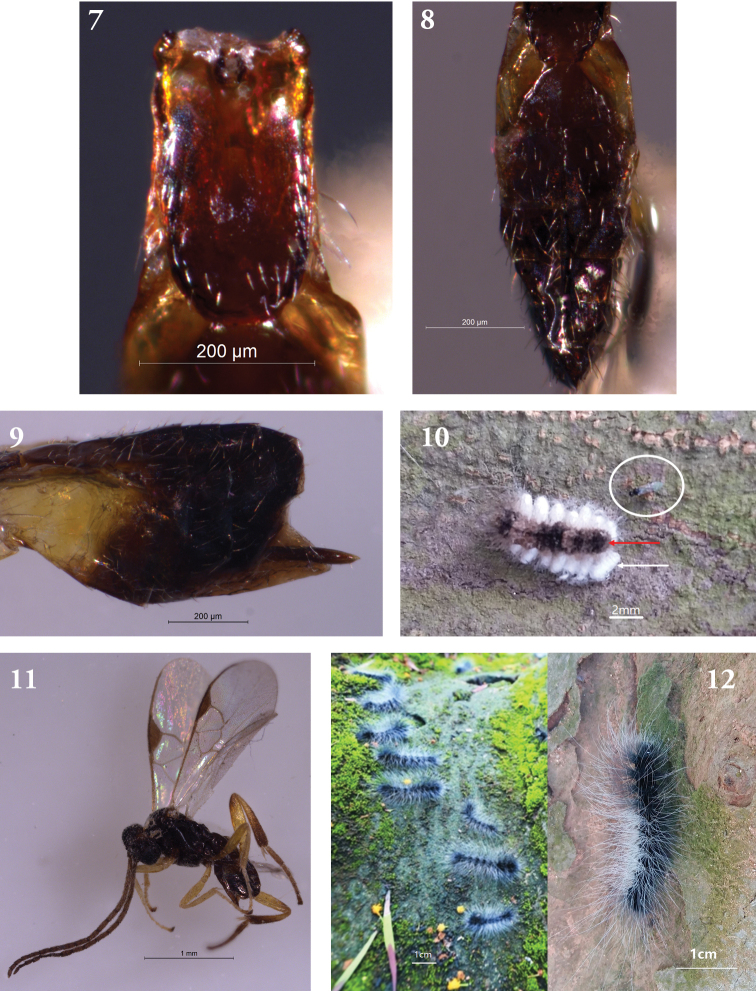
**7–10***Glyptapanteles gigas* Liang & Song, sp. nov. **7** metasoma. T1 smooth, 1.9 times as long as its greatest width, slightly parallel on both sides, base broad depression concave, narrowed toward the end **8** T2 approximately scalariform, the central area inconspicuous with apical width slightly long than central length. T3 1.1 times as wide as long, slightly longer than T2 **9** ovipositor short, ovipositor sheath short, about equal to length of the 2^th^ hind tarsus **10** body mostly black. Antennae black brown. **11** Parasitized larva of *M. gigas*, cocoons and *adult* of *G. gigas*. White ellipse indicating adult of parasitoid. Red and white arrows indicating parasitized larva and cocoons of parasitoid respectively. **12** Unparasitized larvae of the host *M. gigas*.

Metasoma. T1 smooth, 1.9 times as long as its greatest width, slightly parallel on both sides, gradually in general converging apically and rounded to apex, base broad depression concave, narrowed toward the end (Fig. 7). T2 approximately scalariform, the central area inconspicuous with apical width slightly long than central length, T3 1.1 times as wide as long, slightly longer than T2 (Fig. 8). All tergites almost smooth and polished, scattered with feeble setae. Ovipositor short, ovipositor sheath, about equal to length of the 2^th^ hind tarsus with a few hairs on tip (Fig. 9). Hypopygium, evenly sclerotized.

Color. Adult body mostly black (Fig. 10). Antennae black brown; maxillary palps, labial palps, legs yellow, except for most coxae black brown; most hind tibiae and hind tarsus infuscate. Pterostigma dark brown and semi-transparent, most veins pale yellowish. T1 reddish yellow-brown and transparent.

Male. Antenna longer than body (10.0: 7.0), the rest same as female.

#### Remarks.

This new species is closely related to *Glyptapanteles phragmataeciae* (You & Zhou, 1990), but it is easily distinguished from it based on T1 slightly parallel on both sides, gradually in general converging apically and rounded to apex (T1 cuneiform); antenna longer than body (antenna shorter than body); vein 1-R1 1.8 times as long as pterostigma (vein 1-R1 1.0 times as long as pterostigma); inner hind tibial spurs longer than outer one and about half of hind basitarsus (inner hind tibial spurs as long as outer one and shorter than half of hind basitarsus).

##### Hosts

The parasitoid of genus *Glyptapanteles* mostly attacks lepidopteran caterpillars, of which very few species attacks insects of Coleoptera (Nixon 1976; Papp 1990; Smetacek 2008; Tobias 1971, 1976, 1986; Whitfield 1985, 1995, 1997; Whitfield and Wagner 1991; Wilkinson 1936, 1940). Here, we collected parasitoids specimen from caterpillars of *Macrobrochis gigas* Walker (Fig. 11) between 2015-2016 in China, and they occurred in Guangdong, South China (Fang 1985; Taiwan 2019; Mell 1938; Dubatolov et al. 2012; Liu 2005), India, Sikkim, Bhutan, Nepal, Bangladesh, Indonesia (Fang 2000). Taxonomically, it belongs to the family Arctiidae (Telenga 1955; Papp 1983a, 1983b; Fernández-Triana and Ward 2014; Dubatolov et al. 2012), and was firstly recorded and described as a new genera and species in 2001. Biologically, the host insects have one generation per year, and larvae feed on the mosses (Fig. 12) growing on the trunks of masson pines (*Pinus massoniana* Lamb.), litchis (*Litchi chinensis* Sonn.), longans (*Dimocarpus longan* Lour.), coast oak (*Casuarina equisetifolia* Forst.), waxberries (*Myrica rubra* Sieb.et Zucc), eucalyptus (Eucalyptus grandis × urophylla), *Acacia confusa* Merr., and loquats (*Eriobotrya japonica* (Thunb.) Lindl) from April to June, and the moths prefer the flowers after they emerge at the end of July in South China.

## Discussion

In this work, *G. gigas* sp. nov. found in Jinjiang parasitizing caterpillars of *M. gigas* is described and illustrated, and differences from similar species that may parasitize the moss caterpillars are provided. There are 171 species of Lithosiini (Lepidoptera: Erebidae) recorded in China (Fang 1982; Liu 1989; Dubatolov et al. 2012), the majority of which can be found in the rainforests and shrubs on mountaintops, and associated with an abundant food of mosses and lichens found growing on rocks and trees (Liu 1989). Unfortunately, very few parasitoid species are known from those lepidopteran species, partially because host caterpillars usually have an ecological function rather than cause economic losses, and therefore attract very little attention by entomologists; moreover, little is known regarding the trophic relationships and ecological interactions between these phytophagous insects and their host bryophytes (Wang and Luo 2001; Davidson et al. 1989). In fact, some species of bryophytes such as *Brachythecium rutabulum* (Jacek and Adam 2018), *Mnium hornum*, *Trichocoleopsis cacculatta*, *Chiloscyphus polyanthus*, *Wiesnerella denudata*, *Frullania dilatata*, *F. tamarisci*, and *Gymnocolea inflata* were reported to produce a variety of toxic chemicals which repel caterpillars such as *Spodoptera littoralis* and Limacodidae (Asakawa 1990a, 1990b, 1994; Davidson et al. 1989; Shang and Yan 2014). Some other caterpillars found in these relatively ancient habitats have a long co-evolving interaction between insects and lower host bryophytes (Ren et al. 2008).

The moth is possibly involved in a mimicry relationship with *Eterusia aedea* (Linnnaeus, 1763) (Lepidoptera, Zygaenidae) (Fang 2000), which feeds on woody plants of the families Ebenaceae, Fagaceae, Euphorbiaceae, Rosaceae, and Rutaceae, many of which have pyrrolizidine as their main defensive chemical substance (Dubatolov et al. 2012). This phenomenon is similar to that of bracken ferns, which are consumed by some insect species (Hummel et al. 2008; Smart and Hughes 1973; Balick et al. 1978; Ottosson and Anderson 1983), such as Noctuidae, Tenthredinidae, and *Pteris* spp. (Shang and Yan 2014). Actually, there are some toxic substances within mosses of *Brachythecium rutabulum* and *Mnium hornum*, such as oxalic acid (Gerson 1982), phenolic compounds and ferulic acid, cumaric acids, and gallic acid (Davidson et al. 1989).

On the other hand, we assume this new species and their hosts may play an important ecological role in their trophic interaction between host plants and insects (Wu and Lou 2007). The larva of *M. gigas* is characterized by red crochets and long hairy non-poisonous seta instead of venomous seta. Fortunately, these larvae specifically feed on mosses up and down the trunk on rocks rather than on trees or crops, which probably breaks the sporangia and spreads the spores (Shang and Yan 2014); additionally, some spores may be also spread for a long distance by adult moths after their emergence and, some parasitoids will be attracted by the host eggs or larvae, which may also help the spread of spores. Therefore, we presume that specific interactions were involved in the host plant-herbivore-parasitoid system based on the adaptive strategies and coevolution of tritrophic interactions among *G. gigas*, *M. gigas*, and the mosses.

## Supplementary Material

XML Treatment for Glyptapanteles gigas
